# Community coalitions for smoke-free environments in Armenia and Georgia: A mixed methods analysis of coalition formation, implementation and perceived effectiveness

**DOI:** 10.1371/journal.pone.0289149

**Published:** 2023-08-03

**Authors:** Michelle C. Kegler, Ana Dekanosidze, Arevik Torosyan, Lilit Grigoryan, Shaheen Rana, Varduhi Hayrumyan, Zhanna Sargsyan, Carla J. Berg

**Affiliations:** 1 Department of Behavioral, Social, and Health Education Sciences, Rollins School of Public Health, Emory University, Atlanta, Georgia, United States of America; 2 Georgia National Center for Disease Control and Public Health, Tbilisi, Georgia, United States of America; 3 National Institute of Health, Ministry of Health, Yerevan, Armenia; 4 Intervention Development, Dissemination and Implementation Shared Resource, Winship Cancer Institute, Emory University, Atlanta, Georgia, United States of America; 5 Turpanjian College of Health Sciences, American University of Armenia, Yerevan, Armenia; 6 Department of Prevention and Community Health, Milken Institute School of Public Health, George Washington Cancer Center, George Washington University, Washington, DC, United States of America; University of Business and Technology, ALBANIA

## Abstract

Effective models for aligning public health and civil society at the local level have the potential to impact various global health issues, including tobacco. Georgia and Armenia Teams for Healthy Environments and Research (GATHER) is a collaboration between Armenia, Georgia and U.S. researchers involving a community randomized trial testing the impact of community coalitions to promote smoke-free policy adoption and compliance in various settings. Community Coalition Action Theory (CCAT) was used to guide and describe coalition formation, implementation and effectiveness. Mixed methods were used to evaluate 14 municipality-based coalitions in Georgia and Armenia, including semi-structured interviews (n = 42) with coalition leaders and active members, coalition member surveys at two timepoints (n = 85 and n = 83), and review of action plans and progress reports. Results indicated successful creation of 14 multi-sectoral coalitions, most commonly representing education, public health, health care, and municipal administration. Half of the coalitions created at least one smoke-free policy in specific settings (e.g., factories, parks), and all 14 promoted compliance with existing policies through no-smoking signage and stickers. The majority also conducted awareness events in school, health care, and community settings, in addition to educating the public about COVID and the dangers of tobacco use. Consistent with CCAT, coalition processes (e.g., communication) were associated with member engagement and collaborative synergy which, in turn, correlated with perceived community impact, skills gained by coalition members, and interest in sustainability. Findings suggest that community coalitions can be formed in varied sociopolitical contexts and facilitate locally-driven, multi-sectoral collaborations to promote health. Despite major contextual challenges (e.g., national legislation, global pandemic, war), coalitions were resilient, nimble and remained active. Additionally, CCAT propositions appear to be generalizable, suggesting that coalition-building guidance may be relevant for local public health in at least some global contexts.

## Introduction

Community coalitions, a form of strategic association characterized by multiple sectors of a community working together to achieve a shared goal, are a common approach to health promotion. In the U.S., thousands of coalitions exist to address diverse public health and social goals [1–9]. Ideally, coalitions are action-oriented and operate by actively engaging both organizational representatives and community members in making decisions and jointly implementing collaboratively developed action plans based on a deep understanding of local context. By offering a mechanism to pool diverse perspectives, expertise and resources, coalitions are able to implement multiple complementary interventions that synergistically contribute to the desired community change [1,10]. Within tobacco control, especially in the U.S., local coalitions are considered an integral part of a comprehensive approach and are often innovating new intervention strategies as well as implementing evidence-based interventions [11–15].

Outside of the U.S., one of the largest coalition initiatives is the World Health Organization’s Healthy Cities movement. The model highlights the critical role municipalities play in “*establishing the conditions for health”* [16–19], and encourages diverse resident participation and widespread community ownership [17,20]. With respect to global tobacco control, community coalitions are less prominent. The Framework Convention for Tobacco Control (FCTC) outlines a series of evidence-based intervention strategies, but focuses less on how they are to be achieved and implemented [21,22]. Within global tobacco control, coalitions are discussed most often at the national level, but rarely at the local level where they could potentially help to strengthen compliance with smoke-free legislation, which is known to be less than ideal in some countries [23–26]. There have been, however, a few calls to more deeply engage civil society and local grassroots organizations in tobacco control to build stronger public support for the FCTC articles and related policies [15,27,28].

Research and practice on community coalitions have been synthesized into the Community Coalition Action Theory (CCAT) [1,29]. Briefly, CCAT posits that when coalition processes and structures are functioning well, coalition members will contribute their resources to create collaborative synergy that leads to higher quality assessments and action plans, and implementation of evidence-based and promising interventions. Culturally appropriate and science-based interventions then lead to changes in policies, systems, environments and sustainable programs that can drive population-level outcomes. Coalitions develop through stages and can cycle back through them as new issues or priorities are added, and community context influences all aspects of coalition work, from formation through institutionalization [1]. CCAT has been used to synthesize findings from coalition research across a range of topics from COVID-19 to food environments [30–36]. Considerable evidence supports that various aspects of coalition processes such as communication, shared decision-making and leadership, as well as community context, are associated with member participation, satisfaction and other indicators of intermediate effectiveness [7,37–43]. Few studies, however, have examined the full set of associations along the pathway from coalition processes, to member engagement and collaborative synergy, to effectiveness, especially over time [2,39,44–46].

The current mixed methods study describes formation of 14 community coalitions, implementation of their action plans, and intermediate indicators of effectiveness as part of a community randomized trial evaluating whether multi-sectoral coalitions can be formed in countries with different histories of governance than the U.S. (e.g., countries from the former Soviet Union), and if so, whether they can decrease exposure to secondhand smoke through shifting of community norms and adoption of smoke-free policies. Specifically, the paper examines community coalitions formed to promote adoption of and compliance with smoke-free policies to reduce secondhand smoke exposure in 14 medium-sized municipalities in Armenia and Georgia. These countries are ideal settings to examine these overall aims, not only because of their sociopolitical history, but also because they represent countries with two of the highest male smoking prevalence rates globally and have historically lagged in tobacco control [47,48].

**Fig 1:**
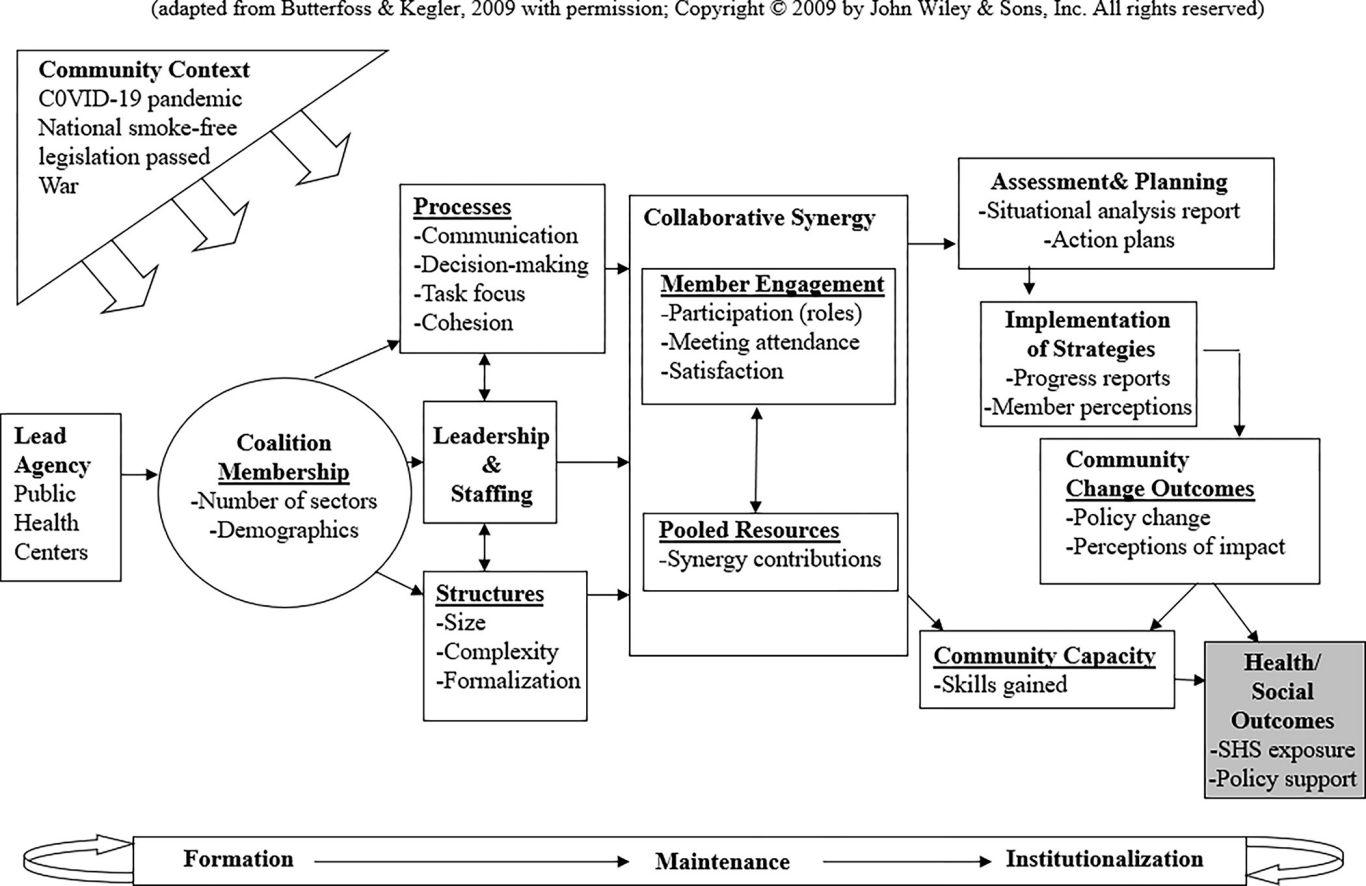
Community coalition action theory operationalized for GATHER.

Specific research questions informed by CCAT (see Fig 1) include: 1) Which community sectors and demographic groups were most likely to join the coalitions? 2) What were the major barriers and facilitators to coalition formation? 3) What settings did coalitions prioritize and what were their major intervention strategies and accomplishments? 4) What contextual factors influenced coalition functioning, implementation and accomplishments? 5) Which intervention strategies were viewed as most effective in reducing SHS exposure? 6) Are associations between coalition processes, member engagement, collaborative synergy, and intermediate outcomes consistent with CCAT predictions?

## Methods and materials

### Tobacco control and COVID-19 context in Georgia and Armenia

Georgia and Armenia Teams for Healthy Environments and Research (GATHER) is a collaboration between the Georgia National Center for Disease Control and Public Health, the National Institute of Health in Armenia, National Centers for Disease Control Armenia, American University of Armenia, George Washington University, and Emory University. The partnership was funded by the Fogarty International Center to build tobacco control research capacity. As part of a community randomized trial, 14 of 28 municipalities were randomized to the intervention group to form a community coalition in 2019. Funds were provided to cover part-time salary support for a local or regional public health professional to form the coalitions and to cover coalition expenses.

At the time the coalitions were formed, both countries had ratified the WHO FCTC over a decade prior and Georgia had just recently (2018) strengthened their national smoke-free policy such that comprehensive smoking restrictions covered a broad range of indoor and outdoor public places, with relatively few exceptions (e.g., parks, mini-stadiums). Armenia strengthened their smoke-free policy in 2020, with implementation of the majority of the provisions in 2022. At the time of the coalition work described here, smoking was still allowed in many public places in Armenia including restaurants and bars, taxis, parks and beaches, hotels, outdoors on school and university grounds, and hotels, and smoking was partially restricted at worksites and playgrounds. Both countries had enforcement practices in place, with compliance monitoring, citations and fines.

**Fig 2:**
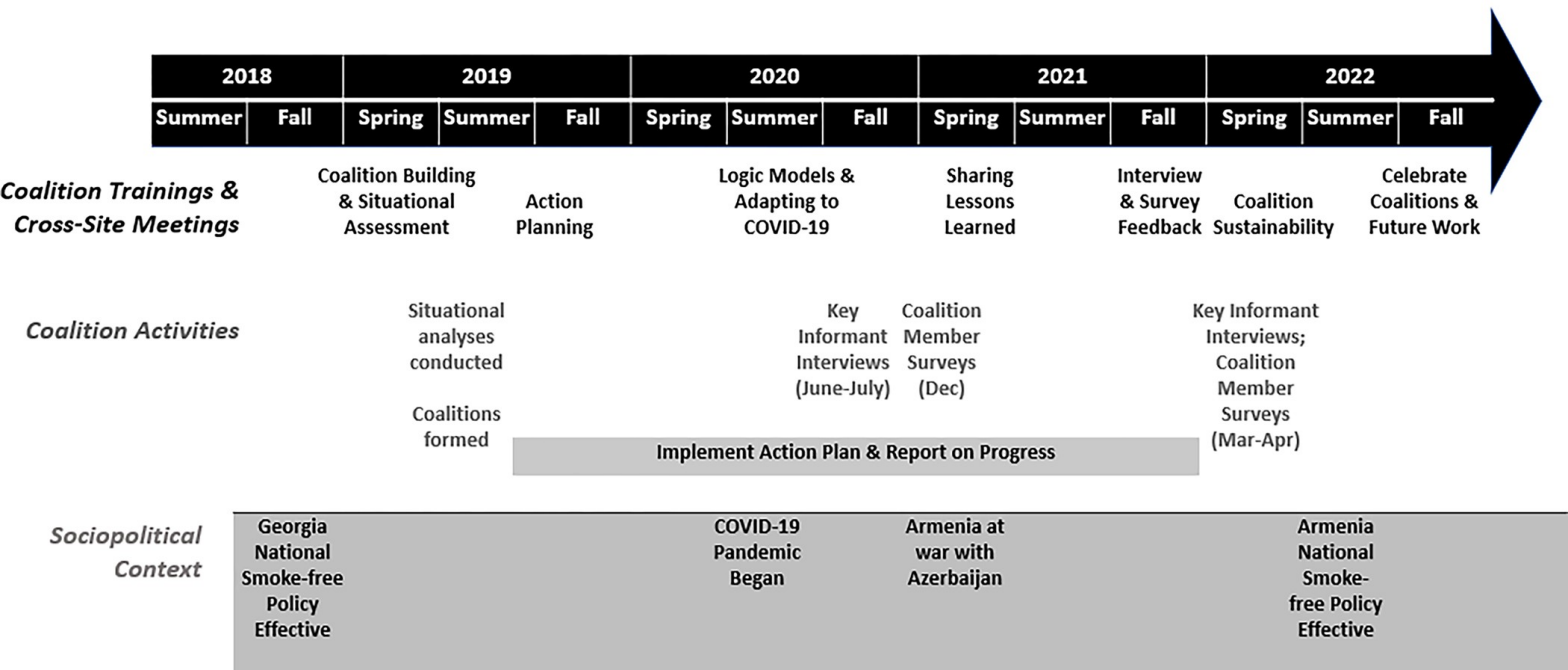
Timeline for GATHER coalition formation and implementation. **Action Planning & Strategy Selection:** Include SMART (specific, measurable, achievable, realistic, and time-based) annual objectives, tasks, timelines, and persons responsible for completing each task. Based on examples of best practices in the US and elsewhere for creating policy change in each type of setting. Steps for policy change include documenting local problems (e.g., observations, key informant interviews), formulating policies (e.g., developing/sharing model policies for different sectors), building awareness (e.g., creating promotional materials, holding awareness and earned media events, developing press releases to media, using social media), and persuading decision-makers (e.g., meeting with decision-makers, encouraging/supporting surveys to assess support for policy change, finding/sharing personal stories, making health/cost savings arguments). Maintain sensitivity to the four step policy-making process (i.e., formulation, enactment, implementation, maintenance).

In this study, coalition leaders were paid staff members. They attended a 1.5 day in-person training in February of 2019 to learn how to conduct a situational analysis for tobacco control (i.e., purpose, key informant interviews, template), and how to form a local coalition (i.e., recruitment, functioning). A second in-person training was held in June 2019 on developing an action plan (i.e., SMART objectives) and maintaining a community coalition. Coalitions began implementing their action plans in Fall of 2019 and were fully operational about six months before COVID-19 began to influence their planned implementation activities. Coalitions were able to continue through the pandemic, but planned activities shifted to accommodate local restrictions (e.g., social distancing, closed businesses, remote learning in schools). Coalitions implemented action plans through 2021, for a total implementation phase of 27 months. See Fig 2 for a timeline of the project, which also calls out timing of contextual factors, including national policy implementation, the COVID-19 pandemic, and the war between Armenia and Azerbaijan.

### Data collection methods, measures and analysis by data source

Three types of data were used in the evaluation: interviews with coalition leaders and an active coalition member, program documents including action plans and progress reports, and coalition member surveys at two points in time. The protocol was reviewed and approved by the Emory University Institutional Review Board, the National Academy of Sciences of the Republic of Armenia Institutional Review Board, the Institutional Review Board of the American University of Armenia, and the National Center for Disease Control and Public Health of Georgia Institutional Review Board. We obtained written informed consent for the key informant interviews and a waiver of documentation of consent for the web-based coalition member survey.

#### Key informant interviews with coalition leaders and active coalition members

Semi-structured interviews were conducted with local coalition leaders in Summer 2020, about one year after coalition formation and action plan development. The interview guides were adapted from prior evaluations of community coalitions [3,43] and covered: prior tobacco control efforts, the situational analysis, coalition formation, membership criteria, coalition structure, staffing, member involvement, the action planning process, implementation, early outcomes and accomplishments, and facilitators and barriers to coalition formation and action plan implementation.

A second round of interviews were conducted in Spring of 2022 after completion of the implementation phase. Local leaders were interviewed, along with the most active member per coalition, as identified by the leader. The interview guides covered: member involvement and contributions, action plan implementation, barriers to implementation, important outcomes including policy change, pros and cons of coalitions as a public health strategy, and interest in sustaining their coalitions. In both rounds of data collection, interviews took 45–60 minutes, were conducted in-person in Georgia and by video conference in Armenia, and audio-recorded.

Qualitative interviews were transcribed verbatim, then translated into English. Members of the Emory team developed a codebook based on the interview guides and review of several transcripts, and then double-coded the interviews. Qualitative data were managed in NVivo 12. Reports were generated for each major NVivo code, and themes were identified. Teams in Armenia and Georgia were involved in identifying and confirming themes, with matrices used to assess strength of theme, similarities and differences across countries, and to provide an audit trail to increase trustworthiness of the findings [49,50].

#### Program documents

The evaluation included two types of document reviews: annual action plans and progress reports. An action plan template was provided and included a menu of 13 settings for policy-related efforts (e.g., restaurants, cultural facilities, public transportation, parks). Coalitions were asked to select their priorities, and then indicate whether their efforts would focus on creating/strengthening policy or enforcing it. For each selected priority setting, the coalitions developed a measurable annual objective, with an accompanying description of tasks, timeframe and who was responsible for completing each task.

Similarly, progress reports were structured to focus on the prioritized settings, along with coalition membership. Specifically, the reports included a list of coalition members with sector represented and date of joining the coalition, and a list of coalition meetings with the number in attendance and a brief summary of the meeting. Additionally, the reports requested a listing of the major activities and progress for each of the prioritized settings, as well as a summary of significant accomplishments, challenges, and technical assistance needs. Coalitions were asked to attach any relevant materials such as presentations and photographs of major events. Timing of the progress reports varied slightly by country, but all coalitions submitted three over the course of the project. Annual action plans and progress reports were submitted to the National Institute of Health in Armenia and the National Center for Disease Control and Public Health in Georgia as part of the GATHER project reporting requirements.

All progress reports and annual action plans were translated into English, imported into NVivo 12, and coded into broad categories (e.g., meetings, policy settings, annual objectives, most significant accomplishment) by one member of the Emory team. Code reports were generated for the settings targeted by the coalitions, and abstracted by one analyst to document which coalitions prioritized which settings, along with a description of the policies adopted. These were then summarized by both setting and coalition, with review and confirmation from teams who provided technical assistance to the coalitions in Armenia and Georgia.

#### Coalition member surveys

The first coalition member survey was conducted from December 2020 to March 2021, about 15 months into the implementation phase (T1). Coalition leaders provided e-mail addresses to the study team, who then sent a web-based survey link to coalition members, followed by up to four reminders. Overall the response rate was 85.9% (85 of 99), with coalition-level responses ranging from 66.7% to 100% (13 of 14 coalitions ≥ 70% response rate). The second was conducted from April and May 2022, following the almost 2.5-year implementation phase (T2), using the same approach. Overall response rate was 85.6% (83 of 97), with coalition-level response rates ranging from 33% to 100% (12 of 14 coalitions ≥ 75% response rate. Results were shared with coalitions at both time points.

The coalition surveys were adapted from previously-published surveys examining coalitions [37,38,40,42,51]. Items were translated into both Armenian and Georgian languages, backtranslated, and in a few instances, modified for clarity. Consistent with CCAT, measures are described below in five broad categories: coalition membership, coalition functioning, member engagement and collaborative synergy, intermediate indicators of effectiveness, and contextual influences.

*Coalition membership*. Broad *sector representation* was assessed through a coalition-level measure of the number of sectors represented on the coalition [37]. The survey included a list of 16 sectors with several adapted for local context (e.g., nongovernmental organization instead of community-based organization and civic groups separately, local/municipal administration instead of local government) and members were asked to indicate the sector they best represented. *Demographic* information included gender, education level and age. Representation and demographic characteristics were assessed similarly at both time points (early in the implementation phase and post-implementation).

*Coalition functioning*: *processes*, *leadership and structure*. *Four coalition processes were measured at each time point*: *communication*, *decision-making influence and method*, *task focus and cohesion*. Frequency and productivity of *communication* among members and between members and staff was assessed on a 5-point semantic differential scale (Cronbach’s α = .94 at T1 and α = .95 at T2), 1 = frequent or productive, and 5 = infrequent, unproductive, respectively. *Decision-making influence* was measured by assessing the extent to which members had influence (1 = a lot of influence, 4 = no influence) on three types of decisions at T1 (e.g., setting goals and objectives for the initiative) and four at T2 (e.g., deciding how to implement specific projects), with Cronbach’s α = .93 at T1, and α = .95 at T2 [37,42]. Items were reverse coded for the initial administration, and assigned differently at the second administration such that higher scores indicated stronger processes.

Four items assessed *task focus* of the coalitions (e.g., there is a strong emphasis on practical tasks in this coalition), with Cronbach’s α = .54 at T1 [37]. One negatively worded item was reworded for the second administration, from “this coalition rarely accomplishes anything concrete” to “accomplishes a lot.” Cronbach’s alpha was .81 for the T2 measure.

*Cohesion* was similarly assessed by averaging four items (e.g., group spirit among member), however the Cronbach’s α indicated the scale was unacceptable, even with deletion of selected items [37]. The question was modified at T2 to avoid negative wording; Cronbach’s alpha became acceptable (Cronbach’s alpha = .79) with the removal of the item “members share a strong commitment to this coalition”.

Because coalition leaders in this study were paid staff, items often used for *leadership and staffing* were combined into a 16-item leadership measure at T1 (e.g., has a clear vision, is respected, works well with coalition members), and reduced to eight items for the second survey [37]. Cronbach’s alpha was .92 and .84, respectively.

Coalition size, a dimension of *structure*, was operationalized as the number of active coalition members, defined as attending at least one coalition meeting in the past year.

*Member engagement (participation and satisfaction) and collaborative synergy*. *Participation* was assessed through *roles played* by coalition members and *meeting attendance* [37]. At the first time point, participants were asked about seven *roles* they might have played on the coalition (e.g., participated in action planning). At the second time point, they were asked about seven roles in the implementation phase. The number of roles was summed for each respondent. *Meeting attendance* was assessed by asking, “Have you attended: almost all of the meetings, some, very few, or none.”

The *satisfaction* measure asked about seven aspects of the coalition (e.g., usefulness of the situational analysis, programs and/or activities selected) at the first time point, and about nine aspects at the end of the implementation phase, with a stronger emphasis on implementation and reach at T2 (Cronbach’s α = .90 at T1 and .92 at T2) [37].

*Collaborative synergy* was assessed with seven items at T1. Sample items included: identify new and creative ways to solve problems, include the views and ideas of all of the partners, implement strategies that are most likely to work in your community, and carry out comprehensive activities that complement each other to produce community change [46]. For each, members were asked: Please think about the people and organizations that are members of your coalition. By working together, how well are these partners [synergy outcome]. Responses (1 = not at all well to 5 = extremely well) were combined to create a scale, with Cronbach’s alpha = .92 at T1. The synergy outcome measure was expanded at T2, with the addition of: break down the work in a way that allows all partners to contribute, achieve outcomes that could not be accomplished by working alone, create feelings of energy, excitement and passion about the work [52]. Cronbach’s alpha for this expanded measure was .97.

*Intermediate outcomes of coalition effectiveness*. To assess *implementation*, coalition members were presented with a list of possible coalition activities within five broad categories: school-based events, signage and stickers promoting smoke-free environments, creating smoke-free policies in specific settings, health care and/or COVID-related messages, and community-based awareness activities. They were asked to indicate which of the activities their coalition completed and allowed to check all that apply. This item was created by the study team and the activities were based on review of progress reports.

For each of the coalition activities implemented, members were asked to indicate their opinion about the *effectiveness* of that activity in contributing to reduced SHS exposure. Response options were: 1 = very ineffective to 5 = very effective. This measure was adapted from the Partnership Self-Assessment Survey [53].

We assessed *skills gained* by coalition members as a measure of strengthened community capacity as new skills have potential to be applied to other community problem-solving efforts. At T2, we assessed 12 skill areas, such as assessing needs and assets, understanding new tobacco control strategies, and funding or mobilizing resources for projects. Members were asked, “for each of the skill areas below, indicate whether your skills have improved 1 = not at all to 4 = a great deal.” Items were summed to create a composite measure of skills gained.

Perceived *community impact* was assessed by asking coalition members the extent to which their coalition brought about improvements in their community such as reduced exposure to SHS and improved compliance/enforcement of smoke-free policies [54]. Response options were 1 = not at all to 4 = a great deal.

*Contextual influences*. Given most of the coalition efforts were conducted in the midst of the COVID-19 pandemic (see Fig 2), we assessed member perceptions of the impact on coalition functioning, implementation, and community views of smoking. For functioning, we assessed frequency of meetings, communication in meetings, and participation levels, with response options of 1 = decreased greatly, 3 = no change, 5 = increased greatly. For implementation, we assessed impact on action plan implementation, innovation in outreach methods, changed coalition priorities and activities, and shifted own priorities away from coalition work, with response options of 1 = decreased greatly, 3 = no change, 5 = increased greatly for the first two items, and 1 = not at all to 4 = a great deal for the latter two items. For community views, we asked about community interest in smoking and impact on attitudes toward smoking, with response options of 1 = not at all to 4 = a great deal.

*Coalition member survey data analysis*. Coalition member survey data were analyzed descriptively using SPSS 26.0. Descriptive results are reported with both countries combined and by country for most constructs, given the different tobacco control contexts (i.e., smoke-free legislation) in the two countries, as well as possible cultural differences that might influence results. Because coalition functioning, engagement and outcome variables were theorized to operate at the coalition level, coalition member responses were aggregated within each coalition to form a coalition-level score. Spearman rank-order correlations were used to examine associations between constructs at the coalition level.

### Mixed methods analysis

Using a convergent triangulation approach, qualitative and quantitative data were combined to provide a comprehensive understanding of coalition formation, implementation and intermediate effectiveness measures (e.g., perceived impact, policy change). Specifically, all three data sources were triangulated to assess coalition structure, implementation, perceived effectiveness, and likelihood of sustainability. To keep results somewhat parsimonious, the richest data sources were used to describe additional aspects of coalition processes and outcomes. Interview data were used to describe coalition formation, the action planning process, and perspectives on a coalition approach. Coalition member survey data were used to document membership composition, coalition processes, and correlations predicted by CCAT. Program records were used to report coalition priorities and policy adoption.

## Results

### Lead agency and staffing

Regional public health branches in Armenia and local public health centers in Georgia served as lead agencies for the coalitions, with paid staff employed by these organizations serving as coalition leaders. All of the coalition leaders in Georgia lived in the community covered by the coalition, the majority did not in the Armenian coalitions. Time devoted to the coalitions and their work varied widely across leaders, from a couple of hours per week to several days in a typical week (Table 1). Leaders generally felt the coalition work aligned with their job responsibilities, with a leader from Georgia stating, “*It is basically the same*, *as I work for the local public health center*”.

**Table 1:** Description of coalition membership at T1 (early implementation), coalition member survey.

Dimension	Total Coalition Members (n = 85)	Armenian Coalition Members (n = 38)	Georgia Coalition Members (n = 47)	Range of Coalition-Level Responses (n = 14)
**Live in community**, n, % yes	81 (95.3%)	35 (92.1%)	46 (97.9%)	66.7% to 100%
**Hours per month**, mean, SD	10.7 (23.71)	6.6 (9.77)	13. (30.13)	2.2 to 48.0
**Meeting attendance**, n, % almost all	49 (57.6%)	18 (47.4%)	31 (66.0%)	0% to 85.7%
**Represent group/organization**, n, % yes	65 (77.4%)	28 (75.7%)	37 (78.7%)	50% to 100%
**Part of paid duties**, n, % yes	15 (18.8%)	1 (2.9%)	14 (30.4%)	0% to 66.7%
**Gender**, n, % female	57 (67.1%)	26 (68.4%)	31 (66.0%)	14.3% to 100%
**Education**, n, % college/graduate school	76 (92.7%)	33 (94.3%)	43 (91.5%)	80% to 100%
**Age,** mean, SD	47.8 (10.42)	50.5 (10.76)	45.7 (9.76)	39.3 to 58.3
**Sector representation**, n, %				
Education	25 (30.5%)	12 (33.3%)	13 (28.3%)	14.3% to 60%
Public health	14 (17.1%)	2 (5.6%)	12 (26.1%)	0% to 50%
Health care facilities/clinics/hospitals	14 (17.1%)	11 (30.6%)	3 (6.5%)	0% to 50%
Local/municipal administration	10 (12.2%)	3 (8.3%)	7 (15.2%)	0% to 33.3%
Nongovernmental organizations	6 (7.3%)	3 (8.3%)	3 (6.5%)	0% to 20%
Private business	3 (3.7%)	2 (5.6%)	1 (2.2%)	0% to 20%
Interested resident	2 (2.4%)	1 (2.8%)	1 (2.2%)	0% to 20%
Criminal justice/safety/police	2 (2.4%)	1 (2.8%)	1 (2.2%)	0% to 25%
Student/child	1 (1.2%)	0	1 (2.2%)	0% to 14.3%
Media	1 (1.2%)	0	1 (2.2%)	0% to 14.3%
Neighborhood group	1 (1.2%)	1 (2.8%)	0	0% to 16.7%
Other	3 (3.7%)	0	3 (6.5%)	0% to 16.7%
**Number of sectors represented**	12	9	11	3 to 5

Note: Missing excluded from denominator.

### Situational analysis as an early coalition-building activity

To begin the coalition-building process, each coalition leader led a situational analysis of smoke-free environments in their communities. Leaders sought to interview representatives from various community sectors and age groups. They most commonly interviewed people affiliated with schools and medical centers, but across the 14 coalitions, they interviewed representatives from municipal government, supermarkets, TV directors, journalists, colleges, pharmacies, hotels, taxi drivers, and general residents. The interviews aided in planning their local efforts and in identifying potential coalition members. A leader from Armenia commented, “*The interviews helped me to select coalition members*, *who assisted me to understand their vision*, *mentality and willingness to work*”.

### Coalition membership

Coalitions averaged seven members. Table 1 lists demographic characteristics of the coalition members, as well as the community sectors represented. Across both countries, the vast majority of coalition members lived in the communities served by the coalition (95.3%). Coalition members generally represented a group or organization (77.4%), but only 18.8% saw the coalition work as part of their paid duties. Education levels were very high with 92.7% reporting a college or graduate degree. The majority were women (67.1%) and the mean age was 47.8 (SD 10.42). Members most commonly represented education (30.5%), health care (17.1%), public health (17.1%), and local municipal administration (12.2%). Media, NGO’s, private business, interested residents, and criminal justice/police were also represented on a few of the coalitions. Church or religious organizations were not represented, nor were housing/property management or social/human services, perhaps because the latter two sectors are not as common as in the U.S. Twelve sectors were engaged across all of the coalitions combined, with nine and eleven sectors represented in Armenia and Georgia, respectively.

Also shown in Table 1 are coalition-level characteristics as assessed through the coalition member survey, with significant variation in composition by coalition. For example, at least one coalition had no members who viewed their time devoted to the coalition as part of their paid duties, and another had mostly men on their coalition (14.3% women) in contrast to at least one with 100% women. Sector representation also varied by coalition with an average of just 3 to 5 sectors represented on any given coalition. At least one coalition reported 60% of its members from the education sector, and another reported just 14.3%.

### Coalition structure

The coalitions had relatively simple structures; just one formed a subcommittee and only one leader described formal operating procedures. A coalition leader in Armenia shared, “*We have operating procedures and specific days designed for meetings*. *Before starting the work in the coalition*, *the members sign an agreement regarding their involvement in the coalition*.*”* There were no formal leadership positions (e.g., volunteer chairperson, secretary). The majority of leaders felt their coalition structures were working well.

### Coalition functioning, member engagement, and collaborative synergy

Table 2 presents descriptive findings on coalition functioning, member engagement and collaborative synergy. In general, coalition members felt they had “some” influence in coalition decisions, felt that communication was productive and frequent, that leaders were competent, and that coalitions had relatively high levels of cohesion and task focus. Median coalition scores are also presented by county, along with range of scores across coalitions. Decision-making influence varied across coalitions from 2.6 to 4.0. Similarly, communication ranged from 3.3 to 4.9, and cohesion and task focus ranged from 2.9 to 4.0 across coalitions.

**Table 2:** Coalition functioning, intermediate outcomes and influence of COVID-19, overall and by coalition, coalition member survey.

Coalition Characteristic	Overall (n = 83)	Coalition-Level (aggregated) Armenia (n = 7)	Coalition-Level (aggregated) Georgia (n = 7)
	Mean (SD)	Median and Range	Median and Range
**Coalition Functioning (T2)**			
Decision-making influence ^a^	3.1 (.60)	3.0 (2.6–4.0)	3.3 (2.7–3.8)
Communication ^b^	4.2 (.95)	3.8 (3.3–4.8)	4.7 (3.9–4.9)
Task focus ^c^	3.6 (.56)	3.4 (2.9–4.0)	3.8 (3.3–4.0)
Cohesion ^c^	3.7 (.47)	3.8 (3.0–4.0)	3.9 (3.6–4.0)
Leadership ^c^	3.6 (.54)	3.5 (3.0–3.6)	3.8 (3.3–4.0)
**Member Engagement & Collaborative Synergy (T2)**			
Satisfaction^d^	3.5 (.48)	3.2 (3.1–3.7)	3.8 (3.0–4.0)
Participation			
Meeting attendance^e^	3.3 (.83)	3.6 (2.5–4.0)	3.1 (2.8–4.0)
Roles played on coalition^f^	4.9 (1.55)	5.3 (3.5–6.5)	4.9 (3.8–5.5)
Collaborative synergy^g^	3.7 (.76)	3.1 (2.3–3.7)	4.2 (3.5–4.8)
**Intermediate Outcomes (T2)**			
Skills gained^h^	3.4 (.55)	3.2 (2.9–3.9)	3.7 (3.2–3.9)
Community impact^h^	3.3 (.54)	3.2 (2.6–3.8)	3.5 (3.3–4.0)
Reduced exposure to SHS	3.3 (.61)	3.0 (2.8–4.0)	3.4 (3.0–4.0)
Influenced/created new smoke-free policies	3.2 (.71)	3.0 (2.2–3.6)	3.4 (3.2–4.0)
Improved compliance/enforcement of smoke free policies	3.4 (.59)	3.3 (2.6–3.7)	3.6 (3.2–4.0)
Increased smoking cessation	3.2 (.67)	3.5 (2.7–3.7)	3.0 (2.6–3.9)
Prevented youth from smoking	3.3 (.80)	3.0 (2.7–3.9)	3.6 (2.2–4.0)
Increased the number of smoke-free homes	3.1 (.71)	2.7 (2.4–3.7)	3.2 (2.9–4.0)
Increased knowledge of SHS harms	3.5 (.67)	3.2 (2.6–4.0)	3.8 (3.5–4.0)
Built public support for smoke-free policies	3.4 (.62)	3.0 (2.6–3.7)	3.6 (3.4–4.0)
Increased knowledge of COVID-19 and tobacco	3.5 (.59)	3.3 (3.0–3.6)	3.8 (3.4–4.0)
Interest in sustainability^i^	3.5 (.74)	3.2 (2.2–3.5)	4.0 (3.1–4.0)
**COVID-19 Impact (T1)**			
On coalition functioning			
Frequency of coalition meetings ^**j**^	1.3 (1.05)	1.5 (1.2 to 1.8)	2.0 (1.6 to 3.9)
Communication in the coalition ^**j**^	2.3 (.98)	1.8 (1.5 to 2.4)	2.3 (1.8 to 4.3)
Participation levels ^**j**^	2.4 (1.06)	1.8 (1.5 to 2.6)	2.4 (1.7 to 4.1)
On implementation			
Action plan implementation ^**j**^	2.3 (1.16)	1.8 (1.2 to 2.4)	2.5 (1.5 to 4.1)
Innovation in outreach methods ^**j**^	2.5 (1.24)	2.6 (1.4 to 3.8)	2.3 (1.5 to 4.0)
Changed coalition priorities and activities ^h^	2.8 (.77)	2.8 (2.6 to 3.0)	2.7 (2.4 to 3.0)
Shifted own priorities away from coalition work ^h^	2.9 (.81)	3.0 (2.8 to 3.3)	2.7 (2.3 to 3.5)
On community views of smoking			
Community interest in smoking ^h^	2.7 (1.29)	2.7 (1.6 to 3.2)	2.5 (1.7 to 4.7)
Impacted attitudes toward smoking ^h^	2.8 (.89)	2.8 (2.3 to 3.0)	2.9 (2.4 to 3.4)

Note: T1-Early in implementation, T-2 end of implementation

^**a**^ 1 = No influence, 4 = A lot of influence

^**b**^ 1 = infrequent/unproductive, 5 = Frequent/productive

^**c**^ 1 = Strongly disagree, 4 = strongly agree

^**d**^ 1 = very dissatisfied, 4 = very satisfied

^**e**^ 1 = none of the meetings 4 = almost all of the meetings

^f^Sum of 7 roles

^**g**^ 1 = not well at all, 3 = somewhat well, 5 = extremely well

^h^1 = not at all, 4 = a great deal

^i^1 = not at all, 4 = very

^**j**^ 1 = decreased greatly, 3 = no change, 5 = increased greatly.

Member engagement and collaborative synergy results are also in Table 2. Satisfaction was generally high, with variation from 3.1 to 4.0 across coalitions. Meeting attendance was closer to “some” of the meetings than “almost all” and members participated in 4.9 of 7 roles, with considerable variation across coalitions. Collaborative synergy was notably different across countries with a median of 3.1 in the Armenian coalitions and 4.2 in the Georgian coalitions.

### Action planning process

Coalition leaders were asked about their action planning process. When describing the selection of priority issues to address, leaders spoke about the training they received from the initiative, situational analysis results, or specific knowledge and experience of the coalition members as influencing the selection. A leader from Armenia stated, “*We ourselves participated in educational activities*, *the ones in [training site]*, *and the situational analysis was discussed*, *and the topics were chosen based on the situational analysis*.” Other factors included community priorities and needs, and coalition member expertise and experience. Coalitions in Georgia selected up to three priorities, with the majority selecting two. In Armenia, coalitions tended to select more priority areas.

Schools, parks and transportation were prioritized most often. Review of the submitted action plans documented that schools were the most common setting for coalition work (12 coalitions), even though all were covered by the national smoke-free legislation. Parks and public transportation were also common intervention targets (10 coalitions). Other settings were targeted as follows: homes/apartment buildings (8), restaurants/catering facilities (7), hospitals and clinics (6), public places such as stores/beauty salons (6), worksites/factories (5), cultural facilities such as theaters and museums (5), kindergartens (4), universities (4), hotels (2), stadiums/mini-stadiums (2), and playgrounds (2).

### Implementation of the action plans and perceived effectiveness of major intervention strategies

In the first round of interviews, coalition leaders suggested that efforts to promote smoke-free homes and to increase awareness and build support for smoking restrictions, tobacco use prevention, and tobacco cessation were common. Children were often engaged in these efforts, through essay writing, videos, games and flash mobs. Table 3 lists common coalition activities categorized into five domains: school-based events, signage and stickers promoting smoke-free environments, creating smoke-free policies in specific settings, health care and/or COVID-related messages, and community-based awareness activities. Most common activities across all coalitions were giving smoke-free signs or stickers to specific settings such as taxis or hotels and hanging posters about the dangers of SHS. Educating youth and families on the harms of SHS and tobacco use, meeting with decision-makers to gain commitment for a smoke-free policy, design contests, surveys to assess support for smoke-free policies, and distributing information about the national smoke-free law were also very common. Coalition members were asked about the effectiveness of the various activities in reducing SHS exposure. Most activities were viewed as somewhat effective, with the highest effectiveness scores for recognizing/rewarding settings that created a smoke-free policy.

**Table 3:** Common coalition activities and perceived effectiveness, coalition member survey.

Coalition Activity	Number of Coalitions (Armenia/Georgia)*	Perceived effectiveness in reducing SHS exposure	
School-Based Events	13 (6/7)	Mean	SD
To educate youth on the dangers of tobacco to prevent initiation	13 (6/7)	4.1	1.15
To educate youth on the harms of SHS	13 (6/7)	3.9	1.35
Targeting families on the harms of SHS & importance of smoke-free homes	13 (6/7)	3.7	1.26
Competitions for winning designs for no smoking messages	13 (6/7)	3.9	1.45
**Signage and stickers promoting smoke-free environments**			
Giving smoke-free home signs/stickers to families/parents	13 (7/6)	3.6	1.35
Giving smoke-free signs/stickers to specific settings (e.g., taxis, hotels)	14 (7/7)	3.6	1.24
Hanging posters about dangers of SHS (e.g., public transport)	14 (7/7)	3.7	1.21
**Creating smoke-free policies in specific settings**			
Meeting with owners/managers/administrators to gain commitment for a smoke-free policy (e.g., universities, hotels, parks factors, restaurant owners, hospitals, schools)	13 (6/7)	3.8	1.25
Drafting and sharing sample policies for different settings	6 (2/4)	3.7	1.07
Recognizing/rewarding settings that create a smoke-free policy	12 (5/7)	4.2	.93
Conducting surveys/interviews in specific locations to document support for smoke-free policies (e.g., parks)	13 (6/7)	3.7	1.28
Cleaning up parks or stadiums to remove cigarette butts and build support for smoke-free policies	3 (0/3)	3.6	1.42
**Health care and/or COVID-related messages**			
Education on SHS harms in health care settings	12 (5/7)	3.8	1.05
Distributing printed materials on tobacco harms in COVID-related settings (COVID hotels, testing sites)	10 (4/6)	3.9	.95
Distributing COVID-related face shields/masks with anti-tobacco messages (e.g., taxi drivers, police)	12 (7/5)	4.0	.92
Hanging posters about COVID and tobacco in public locations	6 (0/6)	3.7	1.17
**Community-based awareness activities**			
Student flash mobs in community settings to promote awareness of SHS harms	9 (4/5)	3.6	1.26
Sporting events with education on tobacco harms (e.g., wrestling)	5 (0/5)	3.6	1.41
Distributing “give-aways” at community events to promote smoke-free policies	11 (4/7)	4.2	1.27
Distributing information about the national smoke-free law	13 (6/7)	4.1	1.23
Distributing information on smoking cessation support (e.g., Quitline, self-help information)	7 (1/6)	4.2	1.02

*Reported by at least 2 of the coalition members responding to the coalition member survey per coalition, or ½ of members in one coalition with just two respondents.

### Facilitators and barriers to implementation, including contextual factors

When asked what facilitated their success, respondents described responsible and committed coalition members. A coalition leader in Georgia stated, *“Each coalition member is involved in all activities with heart and responsibility*. *This brings success*.” Involvement of key community leaders/members was viewed as another strong facilitator. An active coalition member in Armenia explained, *“I think the fact that people saw quite serious government employees and doctors among us*, *such as the deputy mayor… and we also invited people who were not members of the coalition*, *but they came and attended our events*. *That made people more alert and excited*.*”* A coalition leader in Georgia explained how the involvement of the city hall employee was instrumental, *“He [employee of a City Hall] is a necessary person*, *he helps us and he is there with all his will… assists us in implementation of events*. *For example*, *if we want to plan a sports event*, *we have to agree it with the City Hall*, *in this case he helps us in everything without any problem*.*”* Compelling ideas or topics, involvement of children, use of educational materials and incentives, and competition and awards were also seen as significant facilitators to successful implementation.

Challenges were generally related to COVID-19 and the associated restrictions. When asked to describe any activities that did not go well and the reasons why, representatives from almost all of the coalitions described how COVID impacted their work. As an active coalition member in Armenia explained, *“The main challenge*, *I think*, *was the situation of the country in general*, *that we could have done more*, *but we took into account the psychological state of the people*, *the obligation of people to stay away from each other because of the epidemic and kept ourselves as restrained as possible in our plans*.*”* A coalition leader in Georgia stated, *“Ours was one of the first districts to get closed—the army was standing here*. *The public was so frightened*. *We had an event planned*, *we wanted to hold an art event for children and we couldn’t do it because of the pandemic*. *Students*, *youth*, *when we mentioned before*, *we had planned this activity with them*. *We could not gather people because of the pandemic*.*”*

Other challenges to implementation, discussed primarily by the Armenian coalition representatives, included the weather, some pushback from those the coalitions were trying to reach (e.g., taxi drivers), caution by some organizations about needing approval to collaborate, scheduling and timing barriers, and the war with Azerbaijan which resulted in thousands of displaced people, injuries and deaths. With respect to the war, one coalition leader commented, “*Well*, *of course*, *there are people who say*, *well*, *is this an appropriate time for that*? *We are in a war*, *we are in COVID*. *There were such pessimistic people*. *People who objectively thought so*. *But*, *well*, *it is natural*. *Everyone has their own opinion*.”

Coalition members were asked about the impact of COVID-19 on their work in late Fall 2020 (Table 2). In both countries, frequency of coalition meetings, communication, and participation levels were viewed as decreasing due to COVID-19. Similarly, COVID-19 was viewed as negatively influencing action plan implementation, innovation in outreach methods, coalition priorities and activities, and shifting of one’s owns priorities away from the coalition work. COVID-19 was also viewed as having some influence on community interest in smoking and general attitudes toward smoking.

### Policy-related successes

Much of the policy-related work focused on increasing awareness of the national smoke-free laws and promoting compliance with the laws. The national laws were strengthened right before and after the coalition work creating a rather fluid policy environment and reducing the opportunity for new smoke-free policies at the community level. Nevertheless, the coalitions were able to make some progress.

In Armenia, several factories and café’s, and cultural facilities adopted or strengthened smoke-free policies (e.g., removed designated smoking areas), along with a hotel. Additionally, schools clarified and strengthened their enforcement of smoke-free outdoor spaces, and a broad range of settings (e.g., shopping malls, city halls, taxis, public transport) were encouraged to comply with the law and/or protect nonsmokers from SHS. Even in settings covered by the law, it was often not enforced leaving substantive room for improvement in creating smoke-free environments. All of the coalitions contributed to clarification and better enforcement of school-related smoke-free policies, and in terms of new policies not covered by the national policy at the time, four coalitions facilitated new policies. One coalition facilitated smoke-free policies in a factory, bakery and hotel. A second in a café’, a third in a café and a dairy factory, and a fourth in a café and a mineral water factory.

In Georgia, where a stricter smoke-free policy was in place shortly prior to coalition formation, coalitions focused on enforcement and compliance with existing policies (e.g., signage and stickers) in schools, business establishments, clinics/hospital settings, cultural facilities, municipal buildings, restaurants, shops, and stadiums. Three coalitions achieved new policy outcomes. One coalition’s efforts led to a smoke-free mini-stadium, another led to a smoke-free park, and a third contributed to smoke-free city parks and a smoke-free residential building.

### Major accomplishments and perceived community impact

Coalition leaders and members were asked to describe their most important outcomes. Respondents answered in two ways. One group described a specific tobacco outcome (e.g., increased awareness of SHS harms, impact on smoking cessation, new smoke-free policies and spaces). For example, a coalition member in Georgia elaborated on a policy victory along with increased awareness, “*Smoking was banned in the children’s playground*, *we put up no smoke signs there and this is our achievement*. *Even the fact that a single child tells me that my family no longer smokes is an achievement*. *Something concrete and huge- no*, *not that way*. *Raise awareness—this is the achievement of the coalition*. *We worked faithfully and unanimously*.”

Another group focused on the coalitions, describing a new way of working together and/or new and valuable relationships within the coalition and with key community leaders. A coalition leader in Armenia described, *“The achievement of our coalition consisted in the fact that we were strangers*, *but we got together and became relatives*, *and the team work was very pleasant*, *everyone made their own proposal*, *it was accepted*, *we discussed*, *nothing happened by the decision of one person*, *but together we decided what to do․”*

Table 2 presents coalition member perspectives on the impact of the coalition on a range of possible outcomes. Greatest impact was thought to be on increased knowledge of SHS harms and increased knowledge of COVID-19 and tobacco. Lower levels of impact were perceived for smoke-free homes, smoking cessation, and new smoke-free policies.

### Perspectives on a coalition approach to tobacco control

When asked about the strengths of a coalition approach in public health, coalition leaders and members described how group work is more effective than working alone and how discussion within coalitions makes strategies more effective. A coalition leader from Georgia explained, “*I think that the coalition is good*, *it is very important who will be its members*. *If there are members in the Coalition who are decision-makers*, *then the coalition will work well*, *because many barriers are being removed and time will be saved*. *You don’t have to spend so much time with them to overcome these barriers and depend on their attitude and kindness*. *If they want to work in this direction*, *of course they have influence on the society*.” An active coalition member from Armenia stated, “*They complete each other*, *if you are suggesting something*, *another suggestion accompanies it and it is better than alone*. *According to me group work is better*. *It is not possible for us to do all that work alone*. *Someone manages*, *the other takes pictures*, *the other helps and the work becomes more complete*.” Coalitions were viewed as a structure for like-minded and influential people to work together, and seen as enjoyable and fun, as well as good for networking by some.

### Interest in sustaining the coalitions

Most of the members and leaders interviewed expected or hoped that their coalition would continue, although a few were not sure. A coalition leader in Armenia stated, *“Our coalition already has the experience and completed work*, *it has recognition*, *and there are already established collaborations*. *I think we can think about public health programs*, *in general*, *monitoring programs*, *and the coalition will implement those activities*, *especially*, *when we have experience in working with health programs and there is no need to make discoveries*.*”* A coalition member in Georgia explained, “*We continue to think that the coalition shall continue its work*, *all members of the coalition want to plan events as much as possible and work in the medical facilities and spaces where we worked*, *we are happy to do it and we want to continue like this and be so united*, *we have gathered such a team*, *we liked to work when you are not limited*, *you will plan the activity*, *you will carry it out*, *no one will limit you in this*, *you will do whatever you desire*.”

Several coalition leaders and members, more commonly in Armenia, had greater confidence the coalition would continue if there was something specific for them to work on. A coalition member of an Armenian coalition explained, “*If there are new ideas concerning health*, *it seems to me that everyone will agree to work again within the framework of the coalition*, *especially when the main experienced core has already been formed*․” A few had not yet discussed it, but sensed that the connections between coalition members would continue even if the formal coalition did not.

Coalition member interest in sustaining the coalitions mirrored the coalition leaders’ views (Table 2), with high interest among coalition members in Georgia and some interest among coalition members in Armenia, while coalition-level interest varied.

### Associations between coalition functioning and member engagement/collaborative synergy

Table 4 shows coalition-level correlations between decision-making influence, communication, task focus, cohesion and leadership, several measures of member engagement, and collaborative synergy. Early in the implementation phase, all of the coalition processes (i.e., decision-making influence, communication, task focus and leadership) were significantly correlated with satisfaction and collaborative synergy. They were not significantly associated with roles played on the coalition, but decision-making influence and communication were associated with attendance at coalition meetings. Number of sectors represented on the coalition was associated with both measures of participation (i.e., attendance and roles played), as well as satisfaction.

**Table 4:** Cross-sectional and longitudinal associations among coalition factors, member engagement and collaborative synergy.

	**Member Engagement and Collaborative Synergy Early in Implementation (T1**)			
**Coalition Functioning and Sector Diversity Early in Implementation (T1)**	**Attendance at Meetings (T1)**	**Roles Played (T1)**	**Satisfaction (T1)**	**Collaborative** **Synergy** **(T1)**
Decision-making influence	.648*	.337	.640*	.590*
Communication	.712*	.271	.774*	.724*
Task focus	.372	.316	.740*	.636*
Leadership	.483	.366	.770*	.739*
Sectors	.557*	.570*	.637*	.461
	**Member Engagement and Collaborative Synergy at the End of Implementation (T2**)			
**Coalition Functioning and Sector Diversity Early Implementation (T1)**	**Attendance at Meetings (T2)**	**Roles Played (T2)**	**Satisfaction (T2)**	**Collaborative** **Synergy** **(T2)**
Decision-making influence	.615*	.251	.411	.332
Communication	.828*	.022	.582*	.789*
Task focus	.522	.042	.651*	.717*
Leadership	.551*	.128	.618*	.727*
Sectors	.543*	.109	.514	.514
**Coalition Functioning and Sector Diversity End of Implementation (T2)**				
Decision-making influence	.685*	.119	.622*	.543*
Communication	.648*	.002	.831*	.712*
Task focus	.668*	.334	.625*	.708*
Cohesion	.841*	.137	.627*	.679*
Leadership	.632*	.251	.697*	.604*
Sectors	.015	-.015	.456	.334

*Significant at p < .05; Spearman rank order correlation, n = 14.

Associations between early coalition processes (T1) and indicators of member engagement and synergy at the end of the implementation phase (T2) were similar, with just a few changes. Decision-making influence at T1 was not correlated with satisfaction or collaborative synergy at T2, and the number of sectors was no longer significantly correlated with satisfaction.

At the end of implementation (T2), all of the processes were significantly correlated with attendance, satisfaction and collaborative synergy, but not with roles played. Number of sectors was not significantly correlated with satisfaction and collaborative synergy.

### Associations between member engagement, collaborative synergy and intermediate outcomes

As predicted by CCAT, satisfaction was associated with each of the intermediate indicators of effectiveness, including skills gained, community impact, and interest in sustainability (Table 5). One of the participation measures (i.e., meeting attendance) was associated with most of the intermediate indicators with the exception of community impact. Number of roles played on the coalition, however, was not associated with any of the intermediate outcomes. Collaborative synergy, hypothesized by CCAT to be the bridge between member engagement and improved outcomes, was associated with two of the engagement indicators (i.e., meeting attendance and satisfaction, and with all three of the intermediate outcomes (i.e., skills gained, community impact, interest in sustainability).

**Table 5:** Correlations among member engagement, collaborative synergy and intermediate coalition outcomes, end of implementation (T2).

	Collaborative Synergy	Skills Gained	Community Impact	Interest in Sustainability
Meeting attendance	.615*	.780*	.454	.590*
Roles on coalition	-.196	.278	-.048	-.164
Satisfaction	.864*	.727*	.842*	.742*
Collaborative Synergy	1.0	.793*	.780*	.880*

*Significant at p < .05; Spearman rank order correlation, n = 14.

## Discussion

This paper describes the formation and implementation phases of 14 smoke-free air coalitions in Georgia and Armenia. With both countries formerly part of the Soviet Union, it was unknown whether engagement of community members to drive local community change would be embraced as a public health strategy. Historically, decisions and major initiatives in Armenia and Georgia were driven through a centralized, top-down approach. Additionally, it was uncertain whether associations predicted by CCAT, which was informed heavily by wisdom literature in the U.S., would generalize to a different political context.

Community coalitions were successfully formed and sustained in all 14 municipalities over a three-year period, even in the midst of a global pandemic. Multi-sectoral coalitions completed situational analyses, and developed and implemented action plans. Further, almost all of the associations predicted by CCAT were observed in these coalitions. CCAT predicts that well-functioning coalitions, as assessed by shared decision-making, frequent and productive communication, cohesion and task-focus, lead to higher levels of satisfaction and participation among members [1,29]. In the GATHER coalitions, most of the coalition processes were associated with satisfaction and collaborative synergy both early in the implementation phase and at the end. Other studies have shown similar associations [37,38,44]. Additionally, coalition processes as assessed early in the implementation phase were still associated with satisfaction, meeting attendance and collaborative synergy at the end of implementation. Member satisfaction, meeting attendance and collaborative synergy, in turn, were associated with skills gained, perceived community impact, and interest in sustainability. Prior studies of collaborative synergy have documented associations with coalition processes, but not linked collaborative synergy to outcomes [44–46].

Diversity of coalition membership and the number of roles played on the coalitions did not operate as hypothesized by the theory, but were consistent with prior studies [37]. Diversity of sectors was significantly correlated with satisfaction early in the initiative, but not at end of the implementation phase. Diversity, while valued positively and with potential to contribute to both collaborative synergy and equitable processes, is complex in that it can create challenges in cohesion and other coalition processes, but may still contribute to community changes [37]. In the GATHER coalitions, which were relatively small, diversity was modest with the majority of members highly educated and from just a few community sectors. Roles played on the coalition, which has been used as a measure of participation in other studies, warrants more study as associations are mixed [37,38].

A large number of studies have documented that coalitions are able to influence changes in policies, systems and environments that support health at the local level [2,6,8,55–58]. In the current study, half of the coalitions contributed to at least one new smoke-free policy in a local setting, such as parks, worksites and hotels. Local policy change was the original goal of the GATHER project, however with the passage of comprehensive smoke-free legislation at the national levels during the course of the project, the number and types of new local policies that coalitions could target was reduced, particularly in Georgia whose policy was implemented just prior to coalition formation. Nevertheless, coalitions were active in strengthening awareness and enforcement of the existing legislation in a wide range of settings, from schools to museums to stadiums. Coalitions were forced to be creative in awareness activities given restrictions on use of mass media to avoid contaminating control communities. Thus, many of the awareness activities were done in the schools with youth (e.g., flash mobs, essay contests), likely also influenced by a relatively high proportion of coalition members from the education sector. Studies in the U.S. have shown that population-level outcomes are dependent on whether coalition activities are evidence-based and have sufficient reach into the full population, as well as a sufficiently long timeframe for change to occur [2,41,59–61]. Smoke-free policies themselves are considered evidence-based [15,21,22], but at present there is not a strong evidence base for *how* to create local policies and/or how to encourage enforcement [21,22].

In addition to the influence of national smoke-free legislation, the coalitions adapted to other major contextual factors including the COVID-19 pandemic which necessitated shifting strategies to accommodate social distancing as well as the closure, at least for a few months, of many of the organizations and settings that were targeted for intervention work (e.g., schools, restaurants, workplaces). This, in combination with the national smoke-free legislation, shifted some of their work to COVID and tobacco messaging, and general messaging against SHS exposure and tobacco use, the latter heavily focused on youth and their families. Coalition members felt that their impact was greatest in increasing knowledge about the harms of SHS and health effects of COVID-19 and tobacco. New smoke-free policies, smoking cessation, and smoke-free homes were viewed as areas with less impact. Further complicating an already dynamic context, coalitions in Armenia also experienced a war with neighboring Azerbaijan during this timeframe, which not surprisingly, focused attention away from tobacco control.

While this study had a number of strengths such as the longitudinal mixed methods design and triangulation of data sources, it also had limitations that should be considered in interpreting results. These coalitions were formed in mid-size communities with populations ranging from 5,700 to 48,300, and may not generalize to smaller or larger communities with varying levels of resources for public health. Similarly, coalitions may operate differently across countries and communities with varying levels of historical experience with collaborative local efforts to improve health. Those interviewed as key informants were heavily involved in the coalition work and likely do not reflect perspectives from all coalition members. They may also have been influenced by social desirability, thus portraying the coalitions more positively than warranted. Additionally, while response rates were generally high, coalition members who chose not to respond to the survey may have had very different perspectives which are missing from the evaluation.

### Conclusion

This study showed that community coalitions can be formed in political contexts outside of the U.S., and generate enthusiasm for locally-driven collaborative efforts across multiple sectors. While challenged by major contextual barriers (e.g., national legislation, global pandemic, and war), coalitions were resilient, nimble and remained active throughout the project period. Additionally, given the implementation of national smoke-free policies during the timeframe of this study, opportunities for local policy change were more limited than anticipated. As a result, coalitions focused heavily on community education in a range of settings, with a strong emphasis on youth and their families. Whether local efforts were able to strengthen policy support, decrease exposure to SHS, or promote smoke-free policies in private spaces (i.e., homes) will be assessed in the larger trial. The current study documented that CCAT propositions appear to be generalizable, suggesting that coalition-building guidance may be relevant for local public health in at least some global contexts. Future studies could examine how coalitions are formed, who joins, how they operate and what they accomplish in a range of settings. Although not a focus of this study, a train-the-trainer model generally worked in that partners in the U.S. shared coalition expertise with public health officials at the national-level in Armenia and Georgia, who translated the “how to” of coalition building, as well as developing and implementing action plans to the local level with public health as the convener.

## References

[1] Butterfoss FD, Kegler MC. The Community Coalition Action Theory. In DiClemente R, Crosby R, Kegler M, editors. Emerging Theories in Health Promotion Practice and Research. San Franciso: Jossey-Bass, Inc, 2009.

[2] Kegler MC, Halpin S, Butterfoss FD. Evaluation methods commonly used to assess effectiveness of community coalitions in public health: Results from a scoping review. New Dir Eval. 2020; 165:139–57.

[3] Kegler MC, Hermstad A, Haardörfer R, Arriola KJ, Gauthreaux N, Tucker S, et al. Evaluation design for the Two Georgias Initiative: Assessing progress toward health equity in the rural South. Health Educ Behav. 2022 Mar 19;10901981211060330. doi: 10.1177/10901981211060330 35306908

[4] Lee JP, Lipperman-Kreda S, Saephan S, Kirkpatrick S. Youth-led tobacco prevention: lessons learned for engaging Southeast Asian American youth. Prog Community Health Partnersh. 2012;6(2):187–94. doi: 10.1353/cpr.2012.0022 22820228 PMC3641773

[5] Toumbourou JW, Rowland B, Williams J, Smith R, Patton GC. Community intervention to prevent adolescent health behavior problems: Evaluation of communities that care in Australia. Health Psychol. 2019;38(6):536–44. doi: 10.1037/hea0000735 30998065

[6] Bunnell R, O’Neil D, Soler R, Payne R, Giles WH, Collins J, et al. Fifty communities putting prevention to work: Accelerating chronic disease prevention through policy, systems and environmental change. J Community Health. 2012;37(5):1081–90. doi: 10.1007/s10900-012-9542-3 22323099

[7] Flewelling RL, Hanley SM. Assessing community coalition capacity and its association with underage drinking prevention effectiveness in the context of the SPF SIG. Prev Sci. 2016;17(7):830–40. doi: 10.1007/s11121-016-0675-y 27392783

[8] Soler R, Orenstein D, Honeycutt A, Bradley C, Trogdon J, Kent CK, et al. Community-based interventions to decrease obesity and tobacco exposure and reduce health care costs: outcome estimates from Communities Putting Prevention to Work for 2010–2020. Prev Chronic Dis. 2016;13:E47. doi: 10.5888/pcd13.150272 27055264 PMC4830258

[9] Yarnoff B, Bradley C, Honeycutt AA, Soler RE, Orenstein D. Estimating the relative impact of clinical and preventive community-based interventions: an example based on the Community Transformation Grant Program. Prev Chronic Dis. 2019;16:E87. doi: 10.5888/pcd16.180594 31274409 PMC6638589

[10] Lasker RD, Weiss ES, Miller R. Partnership synergy: a practical framework for studying and strengthening the collaborative advantage. Milbank Q. 2001;79(2):179–205. doi: 10.1111/1468-0009.00203 11439464 PMC2751192

[11] Leeman J, Myers A, Grant JC, Wangen M, Queen TL. Implementation strategies to promote community-engaged efforts to counter tobacco marketing at the point of sale. Transl Behav Med. 2017;7(3):405–14. doi: 10.1007/s13142-017-0489-x 28405905 PMC5645280

[12] Rhoades RR, Beebe LA. Tobacco control and prevention in Oklahoma: best practices in a preemptive state. Am J Prev Med. 2015;48(1 Suppl 1):S6–s12. doi: 10.1016/j.amepre.2014.09.001 25528709

[13] Brown LD, Redelfs AH, Taylor TJ, Messer RL. Comparing the functioning of youth and adult partnerships for health promotion. Am J Community Psychol. 2015;56(1–2):25–35. doi: 10.1007/s10464-015-9730-2 26066568 PMC4620943

[14] Folkerth M, Adcock K, Singler M, Bishop E. Citizen science: a new approach to smoke-free policy advocacy. Health Promot Pract. 2020;21(1_suppl):82s–8s. doi: 10.1177/1524839919883586 31908201

[15] Centers for Disease Control and Prevention. Best Practices for Comprehensive Tobacco Control Programs—2014. Atlanta: U.S. Department of Health and Human Services, Centers for Disease Control and Prevention, National Center for Chronic Disease Prevention and Health Promotion, Office on Smoking and Health, 2014.

[16] Dooris M, Heritage Z. Healthy Cities: facilitating the active participation and empowerment of local people. J Urban Health. 2013;90 Suppl 1(Suppl 1):74–91. doi: 10.1007/s11524-011-9623-0 22125115 PMC3764265

[17] World Health Organization. Implementation Framework For Phase Vii (2019–2024) Of The Who European Healthy Cities Network: Goals, Requirements And Strategic Approaches. Geneva, Switzerland: WHO, 2019. [cited 2023, February 4]. Available from: https://apps.who.int/iris/handle/10665/346087

[18] Barton H, Grant M. Urban planning for healthy cities. A review of the progress of the European Healthy Cities Programme. J Urban Health. 2013;90 Suppl 1(Suppl 1):129–41. doi: 10.1007/s11524-011-9649-3 22714703 PMC3764272

[19] Ashton J, Grey P, Barnard K. Healthy cities—WHO’s new public health initiative. Health Promot Int. 1986;1(3):319–24.

[20] Norris T, Pittman M. The healthy communities movement and the coalition for healthier cities and communities. Public Health Rep. 2000;115(2–3):118–24. doi: 10.1093/phr/115.2.118 10968742 PMC1308699

[21] World Health Organization. WHO Framework Convention on Tobacco Control. Geneva, Switzerland: WHO, 2003. [cited 2023 February 4]. Available from: https://fctc.who.int/who-fctc/overview

[22] World Health Organization. WHO report on the global tobacco epidemic, 2008: the MPOWER package. Geneva, Switzerland: WHO, 2003. [cited 2023 February 4]. Available from: https://www.who.int/initiatives/mpower.

[23] Byron MJ, Cohen JE, Frattaroli S, Gittelsohn J, Drope JM, Jernigan DH. Implementing smoke-free policies in low- and middle-income countries: A brief review and research agenda. Tob Induc Dis 2019;17:60. doi: 10.18332/tid/110007 31582949 PMC6770618

[24] Weishaar H, Collin J, Amos A. tobacco control and health advocacy in the European Union: understanding effective coalition-building. Nic Tob Res. 2016;18(2):122–9. doi: 10.1093/ntr/ntv016 25634938 PMC4710205

[25] Mlinarić M, Hoffmann L, Kunst AE, Schreuders M, Willemsen MC, Moor I, et al. Explaining mechanisms that influence smoke-free implementation at the local level: a realist review of smoking bans. Nic Tob Res. 2019;21(12):1609–20. 10.1093/ntr/nty206 30285126

[26] Anderson CL, Mons U, Winkler V. Global progress in tobacco control: the question of policy compliance. Glob Health Action. 2020;13(1):1844977. doi: 10.1080/16549716.2020.1844977 33190609 PMC7671716

[27] Craig L, Fong GT, Chung-Hall J, Puska P. Impact of the WHO FCTC on tobacco control: perspectives from stakeholders in 12 countries. Tob Control. 2019;28(Suppl 2):s129–s35. doi: 10.1136/tobaccocontrol-2019-054940 31147481 PMC6589457

[28] Lin V. The Framework Convention on Tobacco Control and health promotion: strengthening the ties. Glob Health Promot. 2010;17(1 Suppl):76–80. doi: 10.1177/1757975909358365 20595358

[29] Butterfoss F, Kegler M,. Community Coalition Action Theory: Designing and evaluating community collaboratives. Fourth Edition. In Minkler M Wakimoto P, editors. Community Organizing and Community Building for Health and Social Equity New Brunswick, NJ: Rutgers University Press; 2022.

[30] Ghaffari M, Armoon B, Khoramrooz S, Harooni J. Community Coalition Action Theory: Introducing an interventional application to confronting Covid-19. Community Health Equity Res Policy. 2023;43(2):211–7. doi: 10.1177/0272684X211006549 33858240

[31] Harooni J, Ghaffari M. Moving knowledge to action: applying Community Coalition Action Theory (CCAT) to bus seat belt usage. J Lifestyle Med. 2021;11(1):8. doi: 10.15280/jlm.2021.11.1.8 33763337 PMC7957041

[32] Story CR, Kao WK, Currin J, Brown C, Charles V. Evaluation of the Southern Harm Reduction Coalition for HIV Prevention: Advocacy accomplishments. Health Promot Pract. 2018;19(5):695–703. doi: 10.1177/1524839917742850 29186992

[33] Miller RL, Reed SJ, Chiaramonte D, Strzyzykowski T, Spring H, Acevedo-Polakovich ID, et al. Structural and community change outcomes of the connect-to-protect coalitions: Trials and triumphs securing adolescent access to HIV prevention, testing, and medical care. Am J Community Psychol. 2017;60(1–2):199–214. doi: 10.1002/ajcp.12162 28851064 PMC5678968

[34] Eggert LK, Blood-Siegfried J, Champagne M, Al-Jumaily M, Biederman DJ. Coalition building for health: a community garden pilot project with apartment dwelling refugees. J Community Health Nurs. 2015;32(3):141–50. doi: 10.1080/07370016.2015.1057072 26212466

[35] Flood J, Minkler M, Hennessey Lavery S, Estrada J, Falbe J. The Collective Impact Model and its potential for health promotion: overview and case study of a healthy retail initiative in San Francisco. Health Educ Behav. 2015;42(5):654–68. doi: 10.1177/1090198115577372 25810470

[36] Reed SJ, Miller RL, Francisco VT. The influence of community context on how coalitions achieve HIV-preventive structural change. Health Educ Behav. 2014;41(1):100–7. doi: 10.1177/1090198113492766 23855017 PMC3947250

[37] Kegler MC, Swan DW. An initial attempt at operationalizing and testing the Community Coalition Action Theory. Health Educ Behav. 2011;38(3):261–70. doi: 10.1177/1090198110372875 21393621

[38] Butterfoss FD, Goodman RM, Wandersman A. Community coalitions for prevention and health promotion: factors predicting satisfaction, participation, and planning. Health Educ Q. 1996;23(1):65–79. doi: 10.1177/109019819602300105 8822402

[39] Nagorcka-Smith P, Bolton KA, Dam J, Nichols M, Alston L, Johnstone M, et al. The impact of coalition characteristics on outcomes in community-based initiatives targeting the social determinants of health: a systematic review. BMC Public Health. 2022;22(1):1358. doi: 10.1186/s12889-022-13678-9 35841018 PMC9288063

[40] Brown LD, Feinberg ME, Greenberg MT. Measuring coalition functioning: refining constructs through factor analysis. Health Educ Behav. 2012;39(4):486–97. doi: 10.1177/1090198111419655 22193112 PMC3563297

[41] Brown LD, Feinberg ME, Greenberg MT. Determinants of community coalition ability to support evidence-based programs. Prev Science. 2010;11(3):287–97. doi: 10.1007/s11121-010-0173-6 20352332 PMC2904842

[42] Kegler MC, Steckler A, McLeroy K, Malek SH. Factors that contribute to effective community health promotion coalitions: a study of 10 Project ASSIST coalitions in North Carolina. American Stop Smoking Intervention Study for Cancer Prevention. Health Educ Behav. 1998;25(3):338–53. doi: 10.1177/109019819802500308 9615243

[43] Kegler MC, Rigler J, Honeycutt S. How does community context influence coalitions in the formation stage? A multiple case study based on the Community Coalition Action Theory. BMC Public Health. 2010;10:90. doi: 10.1186/1471-2458-10-90 20178633 PMC2842234

[44] Loban E, Scott C, Lewis V, Haggerty J. Measuring partnership synergy and functioning: Multi-stakeholder collaboration in primary health care. PloS One. 2021;16(5):e0252299. doi: 10.1371/journal.pone.0252299 34048481 PMC8162647

[45] Jones J, Barry MM. Exploring the relationship between synergy and partnership functioning factors in health promotion partnerships. Health Promot Int. 2011;26(4):408–20. doi: 10.1093/heapro/dar002 21330307

[46] Weiss ES, Anderson RM, Lasker RD. Making the most of collaboration: exploring the relationship between partnership synergy and partnership functioning. Health Educ Behav. 2002;29(6):683–98. doi: 10.1177/109019802237938 12456129

[47] World Health Organization. ARMENIA STEPS Survey 2016–2017: Fact Sheet. World Health Organization. [cited 2023 February 4]. Available from: https://nih.am/assets/pdf/researches/00380987c602e3895652446d141f5d7b.pdf.

[48] Georgia National Center for Disease Control and Prevention. Tobacco Prevalence Survey, 2019. [cited 2023 February 4]. Available from: https://ncdc.ge/api/api/File/GetFile/22c5e2c0-36d9-4a08-975c-03fa09d21c2e.

[49] Yin RK. Case study research: Design and methods. Thousand Oak, California: Sage; 2009.

[50] Miles MB, Huberman AM, Saldana J. A methods sourcebook. Thousand Oak, California: Sage; 2014.

[51] Granner ML, Sharpe PA. Evaluating community coalition characteristics and functioning: a summary of measurement tools. Health Educ Res. 2004;19(5):514–32. doi: 10.1093/her/cyg056 15150134

[52] Jones J, Barry MM. Developing a scale to measure synergy in health promotion partnerships. Glob Health Promot. 2011;18(2):36–44. doi: 10.1177/1757975911404762 21596938

[53] Hasnain-Wynia R, Sofaer S, Bazzoli GJ, Alexander JA, Shortell SM, Conrad DA, et al. Members’ perceptions of community care network partnerships’ effectiveness. Med Care Res Rev. 2003;60(4_suppl):40S–62S. doi: 10.1177/1077558703260272 14687429

[54] Kegler MC, Williams CW, Cassell CM, Santelli J, Kegler SR, Montgomery SB, et al. Mobilizing communities for teen pregnancy prevention: associations between coalition characteristics and perceived accomplishments. J Adolesc. 2005;37(3 Suppl):S31–41. doi: 10.1016/j.jadohealth.2005.05.011 16115569

[55] Roussos ST, Fawcett SB. A review of collaborative partnerships as a strategy for improving community health. Annu Rev Public Health. 2000;21:369–402. doi: 10.1146/annurev.publhealth.21.1.369 10884958

[56] Evenson KR, Sallis JF, Handy SL, Bell R, Brennan LK. Evaluation of physical projects and policies from the Active Living by Design partnerships. Am J Prev Med. 2012;43 (5 Suppl 4):S309–19. doi: 10.1016/j.amepre.2012.06.024 23079263

[57] Hahn EJ, Rayens MK, Adkins S, Begley K, York N. A controlled community-based trial to promote smoke-free policy in rural communities. J Rural Health. 2015;31(1):76–88. doi: 10.1111/jrh.12087 25182714

[58] LeBrón AMW, Cowan K, Lopez WD, Novak NL, Ibarra-Frayre M, Delva J. The Washtenaw ID Project: A government-issued id coalition working toward social, economic, and racial justice and health equity. Health Educ Behav. 2019;46(1_suppl):53s–61s. doi: 10.1177/1090198119864078 31549551

[59] Hawkins JD, Oesterle S, Brown EC, Abbott RD, Catalano RF. Youth problem behaviors 8 years after implementing the communities that care prevention system: a community-randomized trial. JAMA Pediatr. 2014;168(2):122–9. doi: 10.1001/jamapediatrics.2013.4009 24322060 PMC3946405

[60] Hawkins JD, Oesterle S, Brown EC, Arthur MW, Abbott RD, Fagan AA, et al. Results of a type 2 translational research trial to prevent adolescent drug use and delinquency: a test of Communities That Care. Arch Pediatr Adolesc Med. 2009;163(9):789–98. doi: 10.1001/archpediatrics.2009.141 19736331 PMC2740999

[61] Feinberg ME, Jones D, Greenberg MT, Osgood DW, Bontempo D. Effects of the Communities That Care model in Pennsylvania on change in adolescent risk and problem behaviors. Prev Sci. 2010;11(2):163–71. doi: 10.1007/s11121-009-0161-x 20020209 PMC4454391

